# A nuclear geochemical analysis system for boron quantification using a focused ion beam

**DOI:** 10.1007/s10967-016-5030-z

**Published:** 2016-09-15

**Authors:** E. J. Charlotta Nilsson, Per Kristiansson, Linus Ros, Nathaly De La Rosa, Mikael Elfman, Ulf Hålenius, Jan Pallon, Henrik Skogby

**Affiliations:** 1 Division of Nuclear Physics, Department of Physics, Lund University, Box 118, 221 00 Lund, Sweden; 2 Department of Geosciences, Swedish Museum of Natural History, Box 50007, 104 05 Stockholm, Sweden

**Keywords:** Boron, Nuclear reaction analysis, Double sided silicon strip detector, Yield, Nuclear microprobe

## Abstract

Ion beam analysis has for decades been used as a tool for geochemical analysis of trace elements using both X-rays (particle induced X-ray emission) and nuclear reaction analysis. With the geoanalytical setup at the Lund Ion Beam Analysis Facility, the boron content in geological samples with a spatial resolution of 1 µm is determined through nuclear reaction analysis. In the newly upgraded setup, a single detector has been replaced by a double sided silicon strip detector with 2048 segments. After optimization, boron content in geological samples as low as 1 µg g^−1^ can be measured.

## Introduction

The chemical element boron is of importance in rather diverse fields, e.g. as a dopant in semiconductors and carbon nanotubes, see e.g. [1, 2] and in BNCT (Boron Neutron Capture Therapy), see e.g. [3]. Boron is also of great interest in geology, due to its presence as a constituent in certain minerals (e.g. the tourmaline group minerals) or as a minor or trace element in various minerals in Earth’s crust and mantle (see [4] and references therein). Analyses of boron concentrations in geological samples can be used as an important tracer for large-scale geochemical transfer processes and can reveal additional information about formation processes. Thus, boron has a tangible impact on geological processes, and may be of greater importance than previously thought [5].

Different micro-analytical techniques for measuring boron exist, among these secondary ion mass spectroscopy (SIMS) and particle-induced gamma-ray emission (PIGE) analysis (see e.g. [6]) as well as nuclear reaction analysis (NRA). Advantages of analyzing boron in geological samples using NRA are e.g. that the technique does not suffer from the matrix effects that are generally pronounced in SIMS [7]. Techniques commonly employed for analysis of boron in geological samples, such as prompt gamma activation analysis (PGAA) and neutron activation analysis (NAA), see e.g. [8–10], can in some cases indeed give even higher sensitivity than the proposed NRA method. However, neither of these techniques provides any lateral resolution, needed for 2D imaging of the sample, thus they are not of interest for this particular case.

Dating back to the first publication in 1995 [11], boron-containing samples, mainly with a focus on geological samples, have been analyzed at the Lund Ion Beam Analysis Facility (LIBAF). The main tool of this rather extensive boron program has been the nuclear reaction ${}^{11}{\text{B}}\; + \; p \, \to \alpha \; + \;2\alpha$ [12], utilizing a beam energy of just below 700 keV as the reaction has a broad resonance (300 mb at 660 keV) here. The three alpha particles emitted from the reaction can easily (as their energies are considerably higher than the elastically scattered incoming proton) be detected and counted as a function of the beam charge collected. During these past two decades of operation, the boron analysis program has undergone constant improvement, driven by a long-term fruitful collaboration between geologists and physicists, and we have mastered the measurement of high boron concentrations. Taking the boron analysis system one step further must consequently mean to optimize it for low boron concentrations. This development has taken us to the point where we are today, where we, as will be presented in this paper, have optimized the analysis system to enable boron analyses in geological samples with a detection level of 1 µg g^−1^.

## Theory

Nuclear reaction analysis [13] utilizes the interaction between a light charged particle of MeV energy and an atomic nucleus for analysis. The interactions can be two types, either be described as a Coulomb excitation where the target nucleus is left in an excited state and the light particle loses energy correspondingly or a harder reaction where the strong force plays an important role and the reaction products are different from the original projectile-beam configuration. Since these reactions are depending on the quantum states of the particles the probability of different reactions are normally very energy dependent and resonant behavior is common. Hence, NRA as an analytical method is isotope specific and particularly sensitive when it comes to analyzing the lighter elements where the Coulomb barrier height is lower. In many cases, depth profiling of these light isotopes is also possible and then either the resonant behavior or stopping effects can be utilized. The nuclear reaction, on which the NRA analysis of boron presented in this work is based, is the ${}^{11}{\text{B}}\left( {p,\alpha } \right)2\alpha$ reaction [12], i.e. where a ^11^B nucleus in the sample reacts with a proton of the incoming particle beam, resulting in the production of three alpha particles. This nuclear reaction has a strong, very broad (300 keV) resonance of 300 mb at 660 keV proton beam energy.

The reaction

$$p\; + \;{}^{11}{\text{B}} \to \alpha_{0} + {}^{8}{\text{Be}},$$

$$p\; + \;{}^{11}{\text{B}} \to \alpha_{1} + {}^{8}{\text{Be}}^{*} ,$$

$${}^{8}{\text{Be}}^{*} \to \alpha_{11} + \alpha_{12} .$$

produces alpha particles with an energy distribution extending to 6000 keV, all of which can be used for analysis, provided that their energy exceeds the energy (about 600 keV) of the elastically scattered protons. A typical spectrum consists of a small, sharp peak to the far right (high energy), corresponding to alpha particles (*α*_0_) leaving the ^8^Be nucleus in the ground state. To the left of the *α*_0_ peak, a broad peak/distribution peaking around 3000 keV and extending to low energies, is a superposition of alpha particles (*α*_1_, *α*_11_ and *α*_12_), originating from the ^8^Be nucleus in its first excited state. *α*_1_ contributes a broad peak whereas *α*_11_ and *α*_12_—that are the result of a disintegration of ^8^Be—contribute a more continuous distribution. Of the two possible reactions between the ^11^B and the proton, only the one resulting in the *α*_1_ has a large enough probability for reaction for it to be feasible to use for analysis of boron content.

## Experimental

### Instrumentation

The boron analysis system is part of the LIBAF, located at the sub-micron beamline, fed with particle beam from the 3 MV single-ended NEC Pelletron accelerator. The main features of the facility have been described previously in e.g. [14, 15]. When, as in this case, running the accelerator (which is designed for 3000 keV) at energies below 1000 keV, one section of the machine needs to be electrically shortcut to optimize the ion beam optics and the stability of the accelerator. During the experiments reported here, the samples to be analyzed were bombarded with a beam of protons with an energy ranging from 500 to 900 keV. Most of the analysis, however, was performed at the resonant energy just below 700 keV. The focused proton beam was scanned across the target, with a beam current during the measurements of typically 5 nA. To acquire a 2D distribution of the boron content, the beam was scanned in typically 128 steps × 128 steps (optimally 512 steps × 512 steps), with a step size of 10 µm (optimally 1 µm) and the beam spot size was typically 8 µm (optimally 1 µm). The state of the art analytical parameters will of course come at the cost of an increase in the analytical time needed. For this kind of application it is relevant with a 5–10 µm beam size since it corresponds to the typical penetration depth in geological material at the current energy.

### Detector

**Fig. 1:**
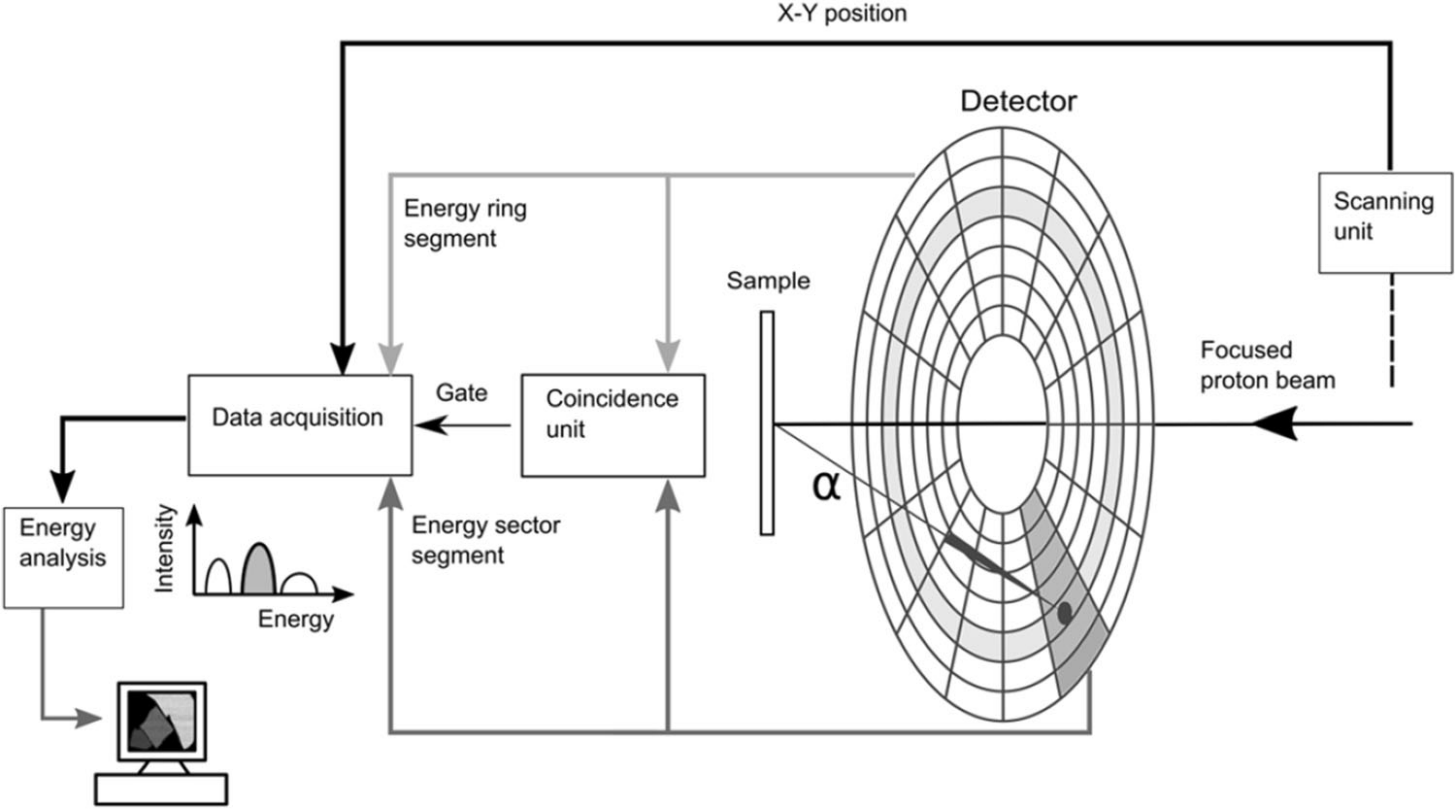
Schematic circuit diagram of the DSSSD setup

A pivotal ingredient in the optimization of the boron analysis system is the upgraded experimental setup, where the single annular surface barrier detector previously used has been replaced by a double sided silicon strip detector, DSSSD, consisting of 2048 segments. The DSSSD has been previously described in detail in e.g. [16, 17]. Of particular interest is the fact that the DSSSD is a strip detector with 64 radial strips on the front side and 32 concentric rings on the back side. Thanks to this large number of independent segments, the DSSSD allows for a much higher beam current to be used, facilitating efficient and essentially pile-up free detection of one of the three alpha particles emitted as a result of the reaction between the incoming proton and the ^11^B in the irradiated sample. The energy calibration of the segments of the detector is conducted using the four energies (482, 554, 976, 1049 keV) from the K and L conversion electrons of a ^207^Bi source. All data presented in this paper has been collected using the DSSSD, in combination with the new Versa Module Europa (VME) based multi-parameter data acquisition and control system [18]. A schematic circuit diagram of the DSSSD setup is shown in Fig. 1.

### Samples and standards

Five different boron containing samples have been used for evaluation of the method and these are as follows: one thin (0.1 µm B on 3.5 µm Mylar) boron standard (Boron Microfoil with 99.6 % purity and ±30 % thickness tolerance from Goodfellow [19]), one thin (200 nm thick layer) boron carbide coated plastic manufactured in-house, one thick sample consisting of three minerals with different B-contents (datolite, tourmaline, axinite; combined DTA standard) provided by the Swedish Museum of Natural History, and the Standard Reference Materials SRM 610 and SRM 612 (from National Institute of Standards and Technology, NIST [20]).

For background measurements, the following different samples have been analyzed: standard and Suprasil synthetic quartz glass, LiF glass, Kapton film [19] and Teflon film [19]. It should be pointed out that the boron measurements are relative, i.e. that a tourmaline or other known boron standard is required. In parallel, a large amount (circa 100) of mineral crystals (mainly clinopyroxene (cpx) and synthetic olivine (ol) crystals) with concentrations varying from 1 to 10,000 µg g^−1^ have been analyzed and evaluated.

### Analytical procedure

The experimental procedure for analysis of a boron containing sample is typically carried out in three steps. Normally, a number of mineral crystals are mounted together as one sample. First, a larger area of the sample is scanned with the beam to find the crystals of interest. Depending on the crystal mounting in the sample, the crystals can either be seen optically or have to be located through backscatter measurements. Once the crystal to be analyzed has been identified, the user interface of the new data acquisition system enables a “custom-made” area of interest to be selected for analysis. This area can assume an arbitrary shape, to focus on special areas inside the crystal or minimizing unnecessary scanning outside the crystal of interest, and consequently optimizing the analysis time. Finally, once all crystals of the sample have been analyzed, the tourmaline crystal, preferably located as one crystal among the others in the sample and necessary for successful internal normalization, is analyzed. The analysis time for the thick combined DTA standard as well as for the thin boron standard was typically 10 min and for the samples used for background evaluation, the analysis time was typically 1 h.

Necessary for successful normalization is also a reliable measurement of the charge delivered to the sample during the experiment. Throughout the experiment, the beam current was sampled pre-target, by deflecting the beam into a Faraday-cup. The pre-sample charge measurement system is described in detail in [21]. Details on the charge measurement are discussed below.

### Energy dependence

An energy scan—i.e. varying the incoming particle energy around the broad resonance—was carried out to further investigate the influence of the proton energy on the boron yield and on the contributions to background. The proton beam energy was varied, from 900 down to 500 keV, in steps of 50 keV. The increment size was narrowed in the most interesting energy interval. For each energy value, three samples—the thick combined DTA and the thin boron standards and the standard quarts—were analyzed in turn. The boron yield, together with the background from ^18^O, was then calculated as a function of proton beam energy.

### Data analysis—extraction of boron

A, to the detector, impinging alpha particle will produce a signal in both one of the sectors and one of the rings, on respective sides of the detector. The energies of these signals are compared in the first step of the data evaluation. This condition pixelated the detector into 2048 pixels which significantly reduces the risk for pile-up. It also filters out signals that have been disturbed by noise in the amplification stage. If several particles enter the detector or the two energy signals do not match, the event is regarded as an invalid event. This is compensated for by reducing the live time of the measurement. In the second stage, the data is sorted by applying an energy window over the desired energy interval from the boron reaction. This filters out most of the unwanted signals from other reactions and is described more in detail in section Results and discussion. In the third stage, the signals are filtered by applying a condition on where on the target the signals where collected. This enables creation of 2D maps of the boron distribution.

To normalise the boron signal yield to the number of incoming protons in the ion beam, the beam is part of the time deflected into a pre-target Faraday-cup, where the beam current is measured using a charge integrator. In this way the charge is collected independent of the structure and conductivity of the sample. The frequency and time needed for charge measurements depends on the beam current used—the lower the beam current, the longer the time needed for collecting an accurate charge value. For the boron measurements the beam was deflected for one second every 10 s. The measured charge value is divided by the time spent on charge measurements giving an estimated current for, in this case, the next 10 s. During this period the analyzing time in each pixel is measured and by multiplying with the current an estimated value of the charge in each pixel is achieved. In this way, this setup provides a sample independent, accurate charge normalization and dead-time compensation. The precision of several measurements on the tourmaline standard are below 3 % using this normalization technique.

Finally the measured boron yield is compensated for the difference in penetration depth (in g cm^−2^) of the proton beam, due to differences in the stopping power of different sample matrixes. This correction factor is calculated using the software “Stopping and Range of Ions in Matter” (SRIM) software and the stoichiometric formula of the sample [22]. This correction is on the order of ±5 % relative to tourmaline for normal geological material.

To achieve the absolute boron concentration, the compensated and, to charge, normalized boron yield is compared to the tourmaline crystal with a boron concentration of 3.27 wt% [5]. This was verified by the use of two NIST-standards with concentration values of 351 µg g^−1^ (information) and 32 µg g^−1^ (information) respectively [20]. In a recent compilation of measurements to determine the boron content in the NIST SRM 610 and 612 standards [23], values in the range (274.5–384) µg g^−1^ for NIST SRM 610 and (32–37.6) µg g^−1^ for NIST SRM 612 are tabulated. The overall, unweighted mean is 350 µg g^−1^ for NIST SRM 610 and 34.3 µg g^−1^ for NIST SRM 612. This spread in the available data is taken into account as an asymmetric error in the comment section of Table 2.

## Results and discussion

**Fig. 2:**
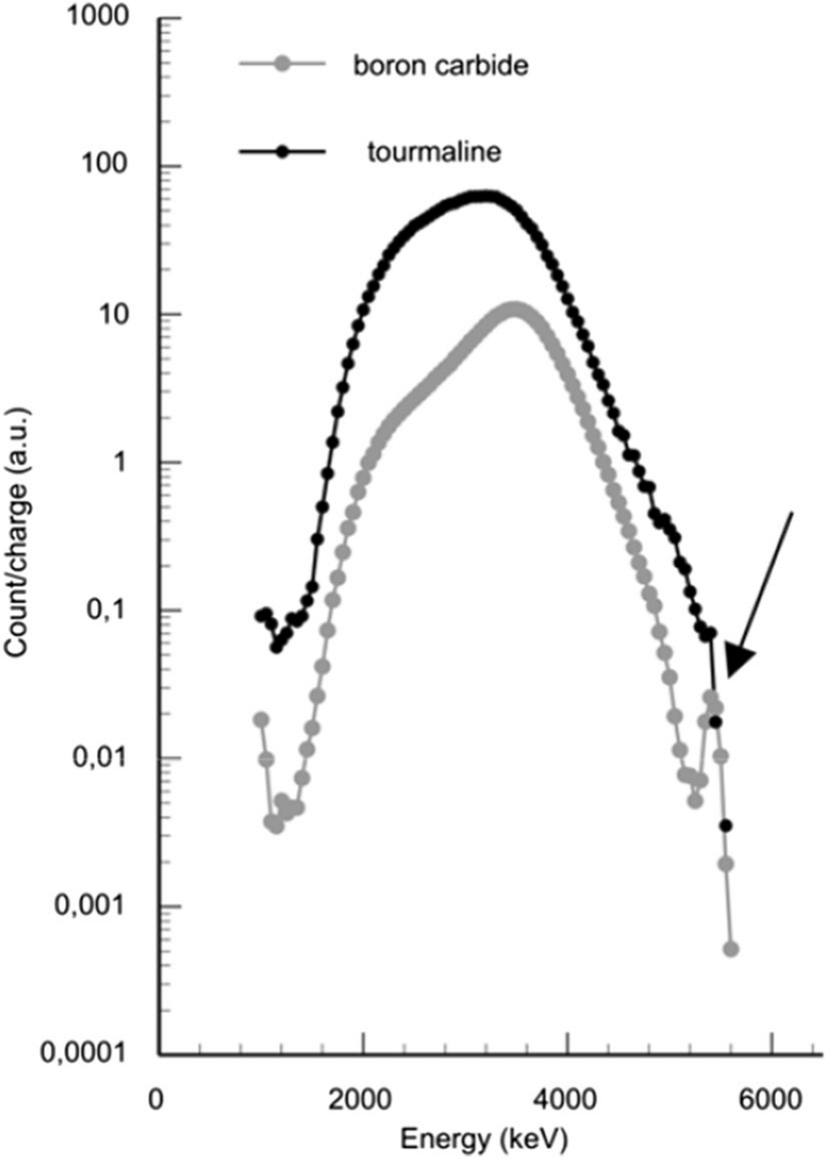
Alpha particle spectra for the nuclear reaction ^11^B(*p*,*α*)2*α* at 600 keV proton energy. Raw data boron signal energy spectra with boron yield as a function of alpha particle energy for thick (DTA tourmaline) and thin (boron carbide) boron samples. Note the *arrow* pointing to the *α*_0_ contribution in the thin sample

Raw data boron signal energy spectra are shown in Fig. 2. In the case depicted, both the thick (DTA tourmaline) and a thin (boron carbide) boron sample were bombarded with a 600 keV proton beam. The yield as a function of measured particle energy is plotted between 1000 and 6000 keV for the two samples. The detected alpha particles resulting from the nuclear reaction ^11^B(p,α)2α can be seen in the figure as the broad distribution between 1900 and 4300 keV. Note that the two curves are not normalized to each other.

The spectra show similar gross features but the tourmaline spectrum has a broader distribution and the maximum value is shifted towards lower energies. This effect originates from that the boron reaction takes place at all different depths but with different probability and in addition, the emitted alpha particles will lose energy on their way out of the sample due to the stopping effect. Stopping or energy loss/unit length originates from the Coulomb interaction between the projectile and the atomic electrons in the sample bulk. The thick sample spectrum can be seen as a folded version of the thin sample spectrum broadened by the stopping effect.

**Fig. 3:**
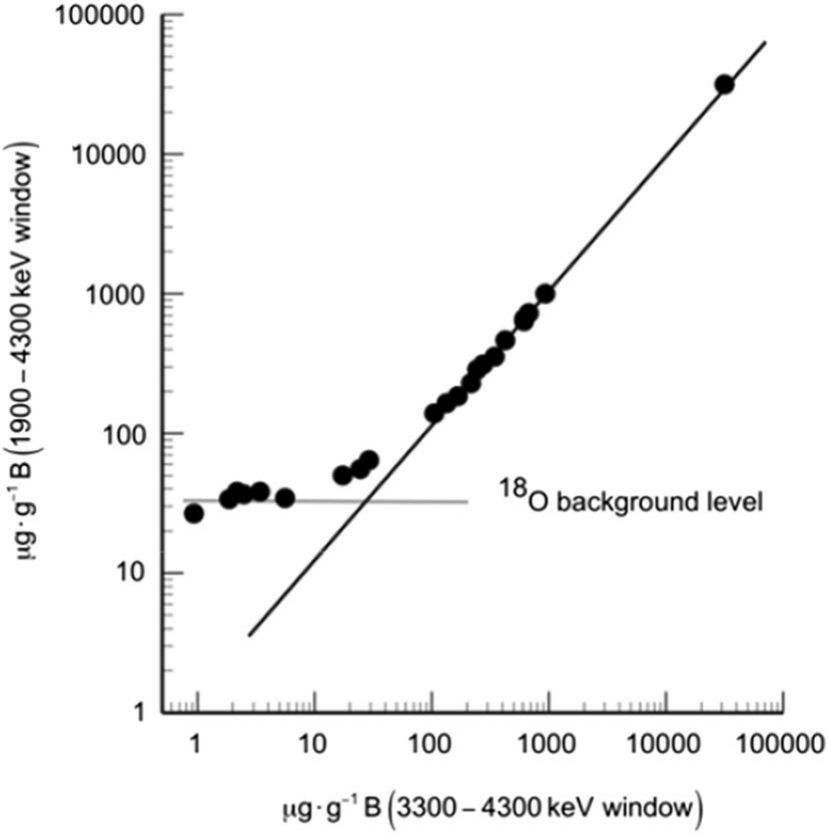
Extracted boron concentrations from the total energy distribution (1900–4300 keV) versus from a high energy window (3300–4300 keV)

Since the boron concentration is deduced from a relative measurement, different parts of the energy distribution can be used. In Fig. 3, the extracted boron concentration from the total energy distribution (1900–4300 keV) is compared with the concentration deduced from a high energy window (3300–4300 keV) for a set of analyzed crystals. The plot shows a linear behavior for high concentrations but going down to lower concentration values, the signal from the broad window flattens out around a 30 µg g^−1^ level. This clearly indicates that there is a non-uniform background present in the spectra that has to be either eliminated or compensated for and that there should be an optimum way to do the quantification at a specific beam energy.

To achieve optimal information from the analysis of a single crystal, possible background sources have to be considered. For the boron analysis three possible sources of background have been considered.

1. Signals from naturally alpha emitting elements with long half-lives either as contamination in the experimental setup or as naturally occurring elements in the geological samples.
2. Pile-up effects in the DSSSD due to the high particle flux during analysis.
3. Nuclear reactions on different isotopes in the crystal to be analyzed.

Point one is easily handled by running the setup without beam before or after the analysis. In the tests performed so far no such contamination has been observed.

Pile-up effects depend both on the current used for analysis and the actual composition of the sample. Since the boron signal is extracted from energy intervals high above the incoming beam energy, the most probable signal will be a combination of a nuclear reaction on ^18^O and a back-scattered beam particle. An estimate of the contribution has been studied on a boron free sample with normal run conditions. From this it has been concluded that the effect corresponded to less than 1 µg g^−1^ boron in the high energy window (3300–4300 keV).

**Fig. 4:**
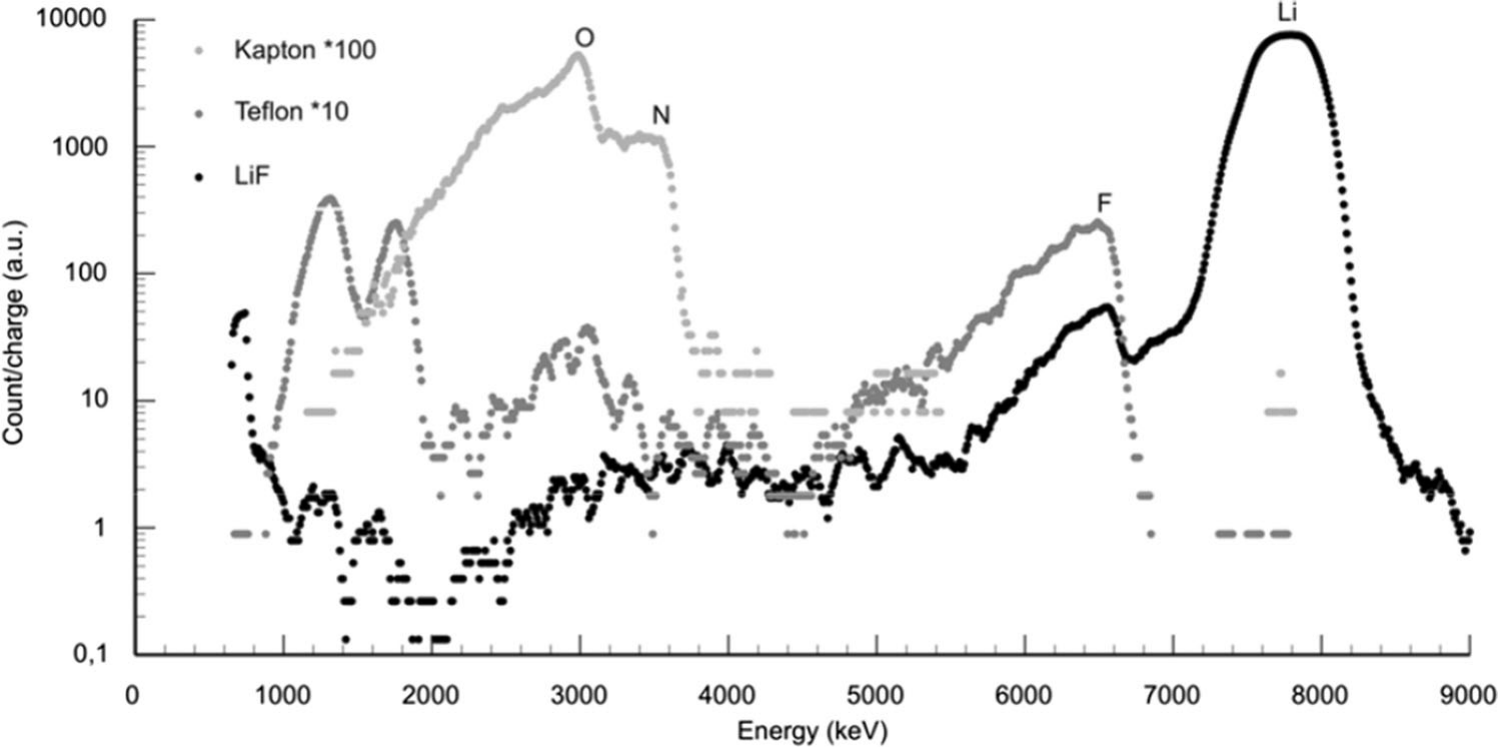
Energy spectra showing contributions from the background elements ^7^Li, ^15^N, ^18^O and ^19^F. The samples analyzed to achieve these spectra were LiF, Teflon (F) and Kapton (N, O)

The most important and limiting effect on the boron analysis is contributions from other nuclear reactions. In Table 1 is listed the *Q* value, i.e. available nuclear energy, and the energy for an alpha particle emitted in 180° for elements between lithium and phosphorus. There are four elements that after reaction can emit alpha particles in or above the, for the analysis, critical energy: ^7^Li, ^15^N, ^18^O and ^19^F. In Fig. 4, energy spectra from measurements of the different background elements are shown and this data has been used to estimate the background in the different relevant energy interval. The samples used are LiF, Teflon (F) and Kapton (N, O).

**Table 1:** *Q* values [24] for the reaction A(p,*α*)B and the corresponding *α*-energy for a proton energy of 600 keV and 150° emission

Isotope	*Q* value (keV)	*E*_*α*_ (keV)
^6^Li	4019	1439
^7^Li	17,346	7951
^9^Be	2126	1273
^10^B	1146	833
^11^B	8688	3α
^15^N	4965	3807
^17^O	1192	1196
^18^O	3979	3325
^19^F	8114	6592
^23^Na	2376	2285
^27^Al	1601	1737
^31^P	1916	2061

Only isotopes with positive *Q* values are included. Reactions overlapping the interesting energy interval for boron analysis are from ^7^Li, ^15^N, ^18^O and ^19^F

In the total energy window analysis, integrating the total boron peak, the totally dominating background comes from ^18^O, and especially since oxygen is a very common element in geological material, this will in general contribute to a background in the order of 100 µg g^−1^ corresponding B_2_O_3_ boron signal. This background could in principle be subtracted based on the knowledge of the chemical composition of the crystal, but will then of course introduce large errors on the estimated content of boron and put limitations on the detection limit.

In the narrow window analysis, the window has been placed above the ^18^O upper energy limit, hence eliminating this background. Then only three interfering elements remain. Lithium and fluorine emit alpha particles with energies much above the relevant interval and the main contributions from those elements are from multiple scattering particles and signal with incomplete charge collection. From the data in Fig. 4 the contributions can be estimated as a function of the yield in the two peaks and for a Li concentration of 1 % a contribution to the boron signal of about 1 µg g^−1^ can be expected. The background generated from ^15^N is more difficult to extract but normally this is not a very common element in geological material. The nitrogen can either be seen directly by the spectral shape or deduced by moving the energy window further up in energy. The yield from this reaction is relatively low and an estimate of the background contribution is less than 1 µg g^−1^ for geological samples.

In summary, there are very small contributions from other processes in the high energy window recommended to be used and a conservative estimate of the background is less than 2 µg g^−1^, which also gives the estimate of the detection limit for B_2_O_3_ in geological samples.

**Fig. 5:**
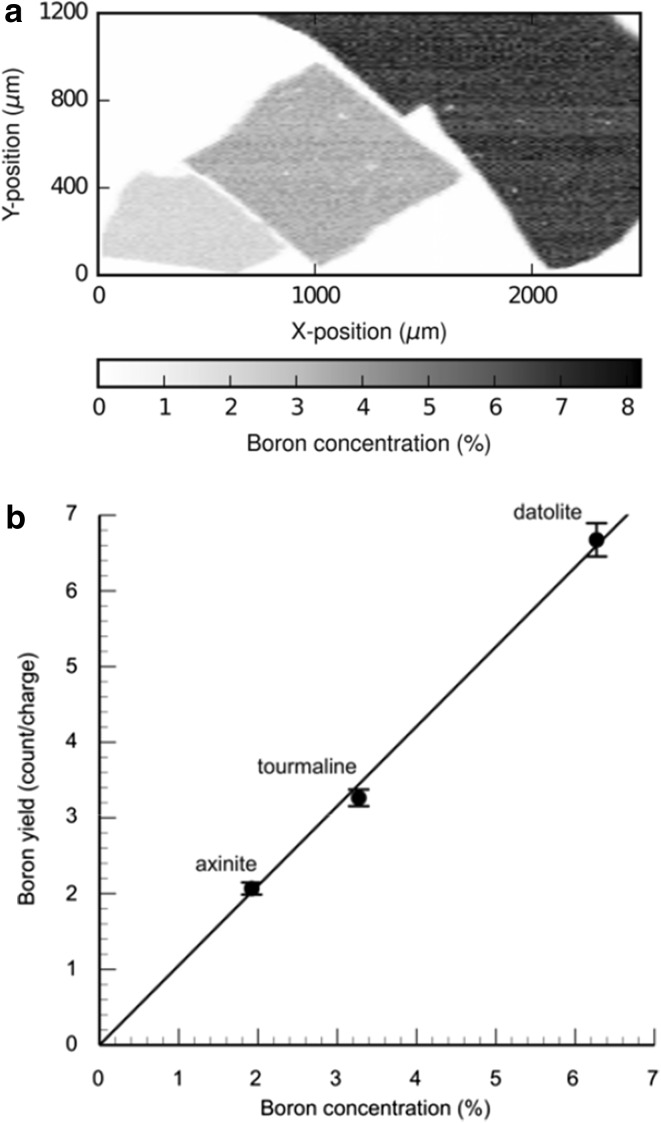
**a**. A 2D map of the combined DTA (from *left to right*: axinite, tourmaline, datolite) standard at proton beam energy 700 keV, (**b**) A calibration curve with corrected yield/charge, in arbitrary units, as a function of the boron concentrations in the DTA standard shown in (**a**). (Color figure online)

In Fig. 5a , a 2D map of the combined DTA standard at proton beam energy of 700 keV is shown. The three different boron concentrations are visible in the map, with the lowest concentration (axinite) at the bottom left of the map. In Fig. 5b is shown a calibration curve with corrected yield/charge, in arbitrary units, as a function of the boron concentration. The three boron concentration values resemble the three different concentrations that are also visible in Fig. 5a (axinite lowest and datolite highest) and the relationship between the two variables is linear. This curve shows that the method is very good for samples with high boron concentration, but for lower concentration values the background has to be considered, and especially important in geological samples is the signal from ^18^O.

**Fig. 6:**
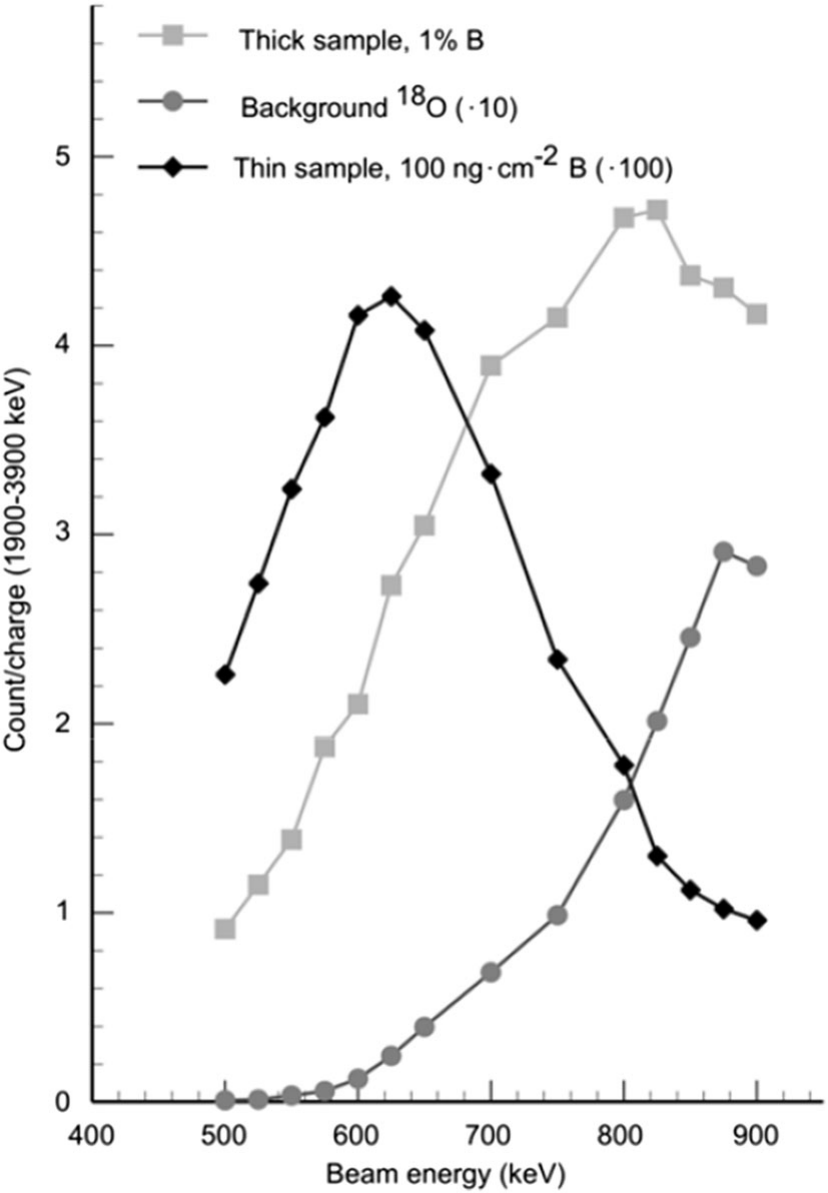
Energy scan plot with boron yield/charge (arbitrary units) as a function of proton beam energy showing the two boron samples [thick DTA tourmaline standard (*squares*) and thin B standard (*diamonds*) together with the oxygen background from the quartz sample (*circles*)] at different proton energies. The thick sample boron content is normalized to 1 % B

The results from the energy scan are shown in Fig. 6. In the figure, the boron yield per charge (in arbitrary units) is plotted as a function of proton beam energy, ranging from 500 to 900 keV, for three different types of samples—thick, thin and blank. An energy window between 1900 and 4300 keV has been used for this data. Shown are the two different boron standards—the thick DTA tourmaline standard (*squares*) and the thin B standard (*diamonds*)—together with the oxygen background from the quartz sample (*circles*) at different proton energies. Here is it clearly seen that the background and the boron yield has different energy dependence and this could be used to optimize which beam energy to be used for a given analysis. At around 600 keV beam energy, the boron yield reaches a maximum for the thin sample, whereas it is about half of its maximum value for the thick sample. The background is significantly decreased at 600 keV beam energy, however, still impeding reaching truly low detection limits.

**Fig. 7:**
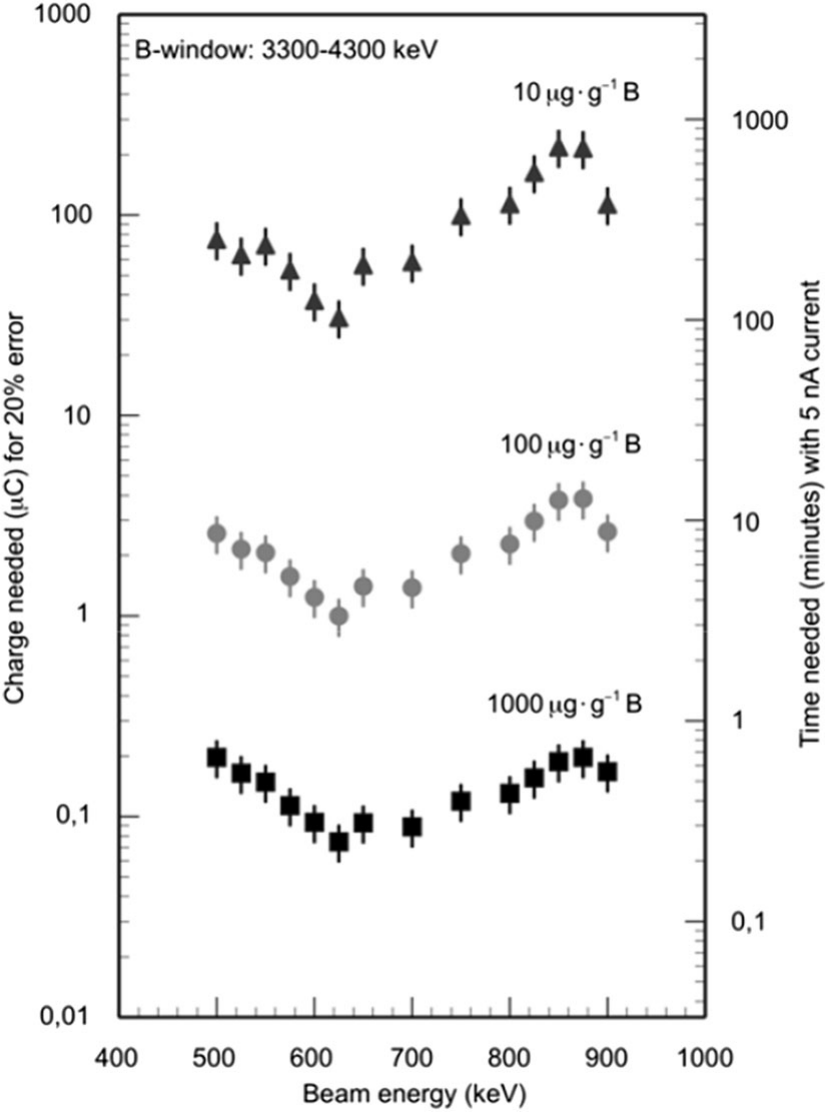
The collected charge needed for 20 % precision as a function of proton beam energy, for three different detection limits (10, 100 and 1000 µg g^−1^). Only the high energy end of the alpha particle energy spectrum has been used. A minimum in charge (or time) needed is found around 620 keV and this minimum becomes more pronounced as the boron content is lowered

The purpose of the optimization activities that have been pursued was to allow for measurement of boron levels as low as 10 µg g^−1^ in geological samples. Since the major source of background comes from ^18^O, even with an optimization of the beam energy, and the energy of this alpha particle is maximum 3300 keV, the solution is to only use the high energy part of the spectrum. This will on the one hand reduce the boron counting statistics by two-thirds, but on the other hand eliminate the background from ^18^O. The result of such an approach is shown in Fig. 7. In this figure, three different detection limits—1000 µg g^−1^ boron, 100 µg g^−1^ boron and 10 µg g^−1^ boron—have been chosen and the collected charge needed to achieve these different detection limits is given as a function of the proton beam energy. In addition, the analysis time necessary to reach these different detection limits is also indicated (the vertical axis to the right), given a beam current of 5 nA, which is typical for these applications. As can be seen in the figure, by choosing the proton beam energy wisely and extending the analysis time per sample to about 3 h or less, it is indeed quite possible to measure boron concentrations well below 10 µg g^−1^.

In Table 2, a typical run protocol is shown to illustrate the analytical procedure, going from detector counts to boron concentration. The first three columns contain the acquired data: count, charge and live time. The next two are the normalized boron yield with statistical errors assuming Poisson counting statistics and the last two, the B concentration with estimated errors derived from the internal tourmaline standard. In the table are also the two NIST-standards (610 and 612) included, to show the capability of the technique extrapolating from 3 % down to 30 µg g^−1^. In addition to the statistical errors, a background error of 1 µg g^−1^ and an error from the charge measurement estimated to 3 % are included in the error estimation.

**Table 2:** Boron data run protocol. Note that the total error is the combination of counting statistics, 3 % charge measurement error and the contribution from the background

	Boron counts (B)	Charge (Q)	LT	B/Q	Error	B	Error	
	Energy region 3300–4300 keV	Pre-sample	Live time (%)	Live time corrected	(Counting statistics)	μg g^−1^	Total in μg g^−1^	Comment
Tourmaline	69,766	114,408	93	0.65404	0.00248	32,687	124	Ref: 3.27 wt% [5]
Tourmaline (run 2)	50,500	82,643	93	0.65721	0.00292	32,845	149	Ref: 3.27 wt% [5]
NIST610	3454	478,968	96	0.00750	0.00013	340	17	Ref: $351_{ - 76}^{ + 33}$ μg g^−1^ B (information, [21])
NIST612	2350	3,197,122	95	0.00077	0.00002	34	2	Ref: $32_{ - 0}^{ + 6}$ μg g^−1^ B (information, [21])
Suprasil (quartz)	16	923,259	100	0.00002	0.00000	0	1	Clean quartz
Quartz	3854	7,362,879	95	0.00055	0.00001	24	2	
Mylar (blank)	27	1,729,600	99	0.00002	0.00000	0	1	
cpx1	164	750,144	91	0.00024	0.00002	0	2	
cpx2	214	1,142,491	97	0.00019	0.00001	8	1	
cpx3	468	1,849,787	82	0.00031	0.00001	13	2	Note: live time
cpx4	45	1,325,579	96	0.00004	0.00001	1	1	
cpx5	82	1,086,664	97	0.00008	0.00001	3	1	N contamination?
cpx6	177	3,661,650	98	0.00005	0.00000	2	1	N contamination?
cpx7	47	1,312,283	98	0.00004	0.00001	1	1	
Synthetic ol1	33,911	350,670	96	0.10047	0.00055	5021	28	
Synthetic ol2	59,570	647,902	97	0.09478	0.00039	4736	20	
Synthetic ol3	31,723	459,221	96	0.07167	0.00040	3581	21	Strong zonation
Synthetic ol4	32,147	355,408	95	0.09473	0.00053	4734	27	High at one edge
Synthetic ol5	52,486	848,979	97	0.06384	0.00028	3190	15	Inhomogeneous
Synthetic ol6	31,564	598,001	96	0.05487	0.00031	2742	16	Zonations high at two edges
Synthetic ol7	82,573	896,991	97	0.09502	0.00033	4748	17	Gradient

From the boron yield measurement on the NIST610 standard, it follows that a concentration of 1 µg g^−1^ corresponds to about 25 counts/h with a 5 nA current. From previous discussion, 1 µg g^−1^ is a conservative estimate of the non-boron related background (i.e. corresponding to 25 counts/h at 5 nA current). As a conclusion, requiring a signal larger than three sigma above background, a detection limit below 1 µg g^−1^ is achieved.

To summarize: the precision in the presented results is limited by the uncertainty in the charge normalization, to 3 %. The accuracy is within the precision of the three standards, i.e. the NIST SRM 610, NIST SRM 612 and the tourmaline samples.

**Fig. 8:**
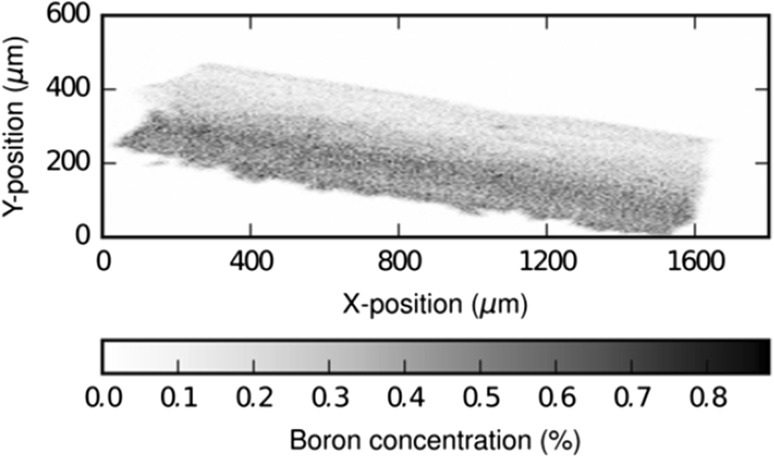
2D boron distribution map of a synthetic olivine crystal (labeled “synthetic ol3” in Table 2), showing clear zonations, normalized to absolute concentrations. (Color figure online)

As an illustration of the strength of this type of analysis, a 2D map of a synthetically produced crystal of olivine with a clear zonation pattern is shown in Fig. 8.

## Conclusions

The development of an analytical technique to determine low boron concentrations in geological material has been described. Different background contributions have been discussed and techniques to reduce or eliminate the influence of their contribution have been demonstrated, leading to an improvement of the earlier technique with detection levels between 10 and 100 µg g^−1^ to new detection levels of close to 1 µg g^−1^.

The main contribution to the background in the standard analyzing procedure comes from oxygen, as can be seen from Fig. 4. Thus, by proper choice of beam energy and by making selective cuts in the energy spectrum (see e.g. Fig. 2), only using the high energy part of the boron spectrum that is situated above the oxygen resonance, the oxygen background can be eliminated and the boron-to-background-ratio can be substantially improved. Analyzing a typical boron-containing geological sample for 3 h with a proton beam current of 5 nA and utilizing this energy window will definitely allow for boron concentrations well below 10 µg g^−1^ to be measured. An estimated lower limit due to background concentration will be around 1 µg g^−1^.

A few words about future activities in the field: Much of the geologically oriented activities at LIBAF during the past 10 years have also included developments in the analysis of isotopic ratios in geological samples. Thus far, techniques for measuring carbon, hydrogen and oxygen isotope ratios [25–27] by various nuclear techniques like scattering, NRA and gamma-tagged NRA (pNRA) [28] have been established and are being utilized regularly. Next, this field will be expanded to also include measurement of boron isotopic ratios (^10^B/^11^B) utilizing a combination of NRA and pNRA techniques. As a spin-off from the background analysis, the investigation of a possible high sensitivity technique to analyze lithium has started. We foresee a possibility to analyze lithium in the 10 µg g^−1^ range, and possibly do simultaneous Li and B analysis for possible Li/B applications.
